# Knowledge, Attitude and Practices of Diabetic Patients in the United Arab Emirates

**DOI:** 10.1371/journal.pone.0052857

**Published:** 2013-01-14

**Authors:** Fatma Al-Maskari, Mohamed El-Sadig, Juma M. Al-Kaabi, Bachar Afandi, Nicolas Nagelkerke, Karin B. Yeatts

**Affiliations:** 1 Institute of Public Health, College of Medicine and Health Sciences, UAE University, Al-Ain City, United Arab Emirates; 2 Department of Internal Medicine, College of Medicine and Health Sciences, UAE University, Al-Ain City, United Arab Emirates; 3 Diabetes Center, Tawam Hospital in Affiliation with John Hopkins Medicine, Al-Ain City, United Arab Emirates; 4 Gillings School of Public Health, University of North Carolina, Chapel Hill, North Carolina, United States of America; Tehran University of Medical Sciences, Iran (Islamic Republic of)

## Abstract

**Introduction:**

Diabetes self-management education is a cornerstone of diabetes care. However, many diabetics in the United Arab Emirates (UAE) lack sufficient knowledge about their disease due to illiteracy. Thus, before considering any possible intervention it was imperative to assess present knowledge, attitudes, and practices of patients towards the management of diabetes.

**Methods:**

A random sample of 575 DM patients was selected from diabetes outpatient's clinics of Tawam and Al-Ain hospitals in Al-Ain city (UAE) during 2006–2007, and their knowledge attitude and practice assessed using a questionnaire modified from the Michigan Diabetes Research Training Center instrument.

**Results:**

Thirty-one percent of patients had poor knowledge of diabetes. Seventy-two had negative attitudes towards having the disease and 57% had HbA**_1c_** levels reflecting poor glycemic control. Only seventeen percent reported having adequate blood sugar control, while 10% admitted non-compliance with their medications. Knowledge, practice and attitude scores were all statistically significantly positively, but rather weakly, associated, but none of these scores was significantly correlated with HbA**_1c_**.

**Conclusions:**

The study showed low levels of diabetes awareness but positive attitudes towards the importance of DM care and satisfactory diabetes practices in the UAE. Programs to increase patients' awareness about DM are essential for all diabetics in the UAE in order to improve their understanding, compliance and management and, thereby, their ability to cope with the disease.

## Introduction

The management of diabetes mellitus (DM) largely depends on patients' ability to self-care in their daily lives, and therefore, patient education is always considered an essential element of DM management. Studies have consistently shown that improved glycemic control reduces the rate of complications and evidence suggests that patients, who are knowledgeable about DM self-care, have better long term glycemic control [1], [2], [3]. Thus it is indispensable to ensure that patients' knowledge, attitudes and practices are adequate.

Although the prevalence of DM is high among populations in the Middle East and Gulf countries, patients often lack the knowledge and skills to self-manage their condition [4]–[7] and although the International Diabetes Federation (IDF) in 2011 ranked the UAE's prevalence for type 2 DM as the tenth highest in the world (19.2%) [8], little is known about the knowledge, attitudes and practices of DM patients in the UAE. In 2006, a study demonstrated poor levels of compliance and knowledge among DM patients in the UAE. Twenty-five percent only of the patients reported an increase in their physical activity levels following diagnosis with a mere 3% meeting the recommended guidelines and 76% could not distinguish between low and high carbohydrate glycemic index food items [9], [10]. To date, only one study assessed DM knowledge among patients in a primary health care setting in the UAE, and identified significant knowledge shortfalls in this population [11]. Since its publication in 2001 there has been considerable media coverage of DM and the level of general education of the population has also increased substantially. A new survey on DM patients' knowledge, attitudes and practice about DM is therefore badly needed.

## Materials and Methods

### Ethics Statement

This study was approved by Al-Ain Medical District Human Research Ethics Committee (MDREC). Informed written consent was obtained from all literate participants while verbal consent was obtained from illiterate participants, for which we obtained approval from Al Ain MDREC. The researchers ensured that the verbal consent contained all the elements of the written consent. The research nurses, in the presence of a witness, explained verbally all the pertinent information of the study and allowed the subjects the opportunity to ask questions and verified that this was understood. Both the research nurse and the witness signed the consent forms when the participants verbally agreed to participate.

### Setting

The study was carried out at the outpatient departments of two major government hospitals, Tawam and Al-Ain hospitals, which serve approximately three quarters of the patients' population in the Eastern District of Abu Dhabi Emirate (Al-Ain region). The health care system in the region is organized along the lines of conventional health care systems, i.e. primary health care (provided by 18 healthcare centres), including basic health care to DM patients, with referral to secondary and tertiary care where needed, provided by the above (only) two referral government hospitals. For logistical reasons (data completeness and accessibility) only referred patients, i.e. those attending the diabetes centres at Al-Ain and Tawam hospitals, were included in the study.

### Study design and selection of participants

The study was a cross-sectional survey to assess the knowledge, attitude and practice (KAP) of diabetic patients in Al-Ain District, UAE using a modified instrument, adopted, with permission, from the Diabetes Research Training Center of Michigan [12]. In addition to KAP, we collected socio-demographic data that include gender, age, occupation, marital status, educational level, income, family history of diabetes, duration of diabetes and medications. The questionnaire was translated into Arabic separately by two bilingual translators. The two versions were combined and revised and then back translated into English by another bilingual translator. The translation was refined after back translation until agreement was obtained among the four people involved in the translations. Two diabetologists examined and approved the Arabic version of the questionnaire for content and construct validity. The questionnaire was then piloted among 10 outpatient DM patients, which gave rise to minor rewordings of the questionnaire. The sampling frame comprised all UAE and non-UAE diabetic patients of all ages and both genders attending the diabetes centres of Al-Ain or Tawam hospitals. In the absence of any diabetes registries, patients were randomly selected from the lists of clinic appointments. We decided that a sample size of *572* would be adequate. This number would provide 90% power, at the 5% significance level, to detect an association between two dichotomous (1/0; y/n) variables, one that splits the sample into two approximately equal halves (e.g. male and female, or the two participating clinics) and another that is 10% and 20% positive for each of the levels of the first variable. To reach this target 620 patients were approached, out of whom 575 (92%) agreed to participate.

### Data collection and definitions

Informed consent was obtained from each patient at the time of their visit to the hospital. Literate patients filled out the questionnaires themselves while illiterate participants were interviewed by trained nurses. Clinical data, including diabetes complications and HbA**_1c_** (within six months prior to the survey) of participants were retrieved from medical records (HbA**_1c_** available for 208 patients only). Since, it was not always possible to distinguish clearly between types 1 and 2 DM from these records, patients were classified as either “insulin treated”, or “non-insulin treated”. Glycemic control was considered good, acceptable or poor when HbA**_1c_** levels were less than 7%, 7 to 8% and greater than 8, respectively, according to the American Diabetes Association's recommended guidelines [13].

### The instrument

In the questionnaire patients' knowledge of diabetes was assessed using 23 questions relating to definitions, symptoms, causes and complications of DM. Attitudes were assessed using a series of questions on positive and/or negative attitudes towards having the disease, the ability to self-manage diabetes and awareness of the importance of adherence to DM (self) care. Patients' practices were assessed using questions on self-care, dietary modification, compliance with medications, weight control, self-monitoring of blood sugar, and regular follow up. DM knowledge was then scored by assigning one point for each correct response. We considered a score of 19–23 ‘Good Knowledge’; a score of 15–18 ‘Moderate Knowledge’ and 0–14 ‘Poor Knowledge’. Attitudes were elicited using Likert scales with 0 = strongly disagree, 1 = disagree, 2 = neutral, 3 = agreement and 4 = strong agreement. Patients' responses were summarized and a score of 1–32 was considered ‘Negative Attitude’ and a score of 33–44 a ‘Positive Attitude’. Similar Likert scales were used to assess patients' practice where a score of 1–8 was considered ‘Negative Practice’ while a score of 9–12 was considered ‘Positive Practice’.

### Statistical analysis

Data were analyzed using SPSS version 19. All statistical tests were performed using 0.05 as the level of significance. One-way ANOVA and Student *t*- test were used to compare groups. Correlation between variables was assessed using Pearson correlation coefficients. Scale properties of the knowledge and attitude scores were assessed using Cronbach's Alpha (as the practice score essentially asked about all essential elements of good practice this was considered inappropriate for this score). Stepwise linear regression analysis was used to examine the simultaneous effect of various patient characteristics on patient knowledge, practice, attitude, and HbA**_1c_** levels.

## Results

Of the 575 participants 55% were females, 65% were UAE citizens and 46% were illiterate. Twelve percent were current smokers. The mean (SD) age of the sample was 50 (15) years and the mean duration of diabetes was 9 (7) years. Mean HbA**_1c_** was 7.7±(3.3)%. Other patients' socio-demographic and clinical characteristics are shown in Tables 1 and 2 respectively.

**Table 1:** Socio-Demographic Characteristics of the Study Participants (n = 575).

Variable	N (%)
**Sex**	
Female	316 (55.1)
**Level of Education**	
illiterate	265 (46.3)
elementary	143 (25)
secondary	105 (18.4)
college	59 (10.3)
**Age group (Years)**	
≤39	114 (19.9)
40–49	138 (24)
50–59	153 (26.7)
60 or above	169 (29.4)
**Nationality group**	
UAE	374 (65.2)
Other Gulf Council countries (GCC) citizens	85 (14.8)
Arabs from other countries	115 (20)
**Marital status**	
Single	59 (10.3)
Married	417 (73)
Divorced	20 (3.5)
Widowed	75 (13.1)
**Monthly family income**	
<5000 Dhs.	208 (36.9)
5000–9000 Dhs.	219 (38.9)
10,000–15,000 Dhs.	101 (17.9)
>15,000 Dhs.	35 (6.2)
**Occupation**	
Government employees	106 (18.6)
Private employees	24 (4.2)
Private business	4 (0.7)
Retired	120 (21.1)
Housewives	279 (49)
Students	36 (6.3)
**Place of interview**	
Tawam Hospital	299 (52)
Al-Ain Hospital	276 (48)

**Table 2:** Clinical Characteristics of the Study Participants (n = 575).

Variable	Proportion of all Diabetics	
	N	Percent (95% CI)
**Type of DM**		
Insulin treated diabetes	198	34.9 (30.98–38.82)
Non-insulin treated diabetes	370	65.1 (61.18–69.02)
**Mode of diagnosis**		
Incidental	189	34.5 (30.52–38.48)
Symptomatic	359	65.5 (61.52–69.48)
**Family history of DM**		
Present	360	64.4 (60.43–68.37)
**Duration of DM**		
<1 year	47	8.5 (6.18–10.82)
1.1–5 years	143	25.8 (22.16–29.44)
5.1–10 years	151	27.2 (23.50–30.90)
10.1–20 years	183	33 (29.09–36.91)
>20 years	31	5.6 (3.69–7.51)
**Other chronic conditions**		
Present	317	61.2(57.00–65.40)
**Glycemic control**		
Good (HbA**_1c_** <7%)	74	26.9 (21.7–32.1)
Acceptable (HbA**_1c_** 7–8%)	45	16.4 (12.0–20.8)
Poor (HbA**_1c_** >8%)	156	56.7 (50.8–62.6)

### Knowledge Assessment

**Figure 1:**
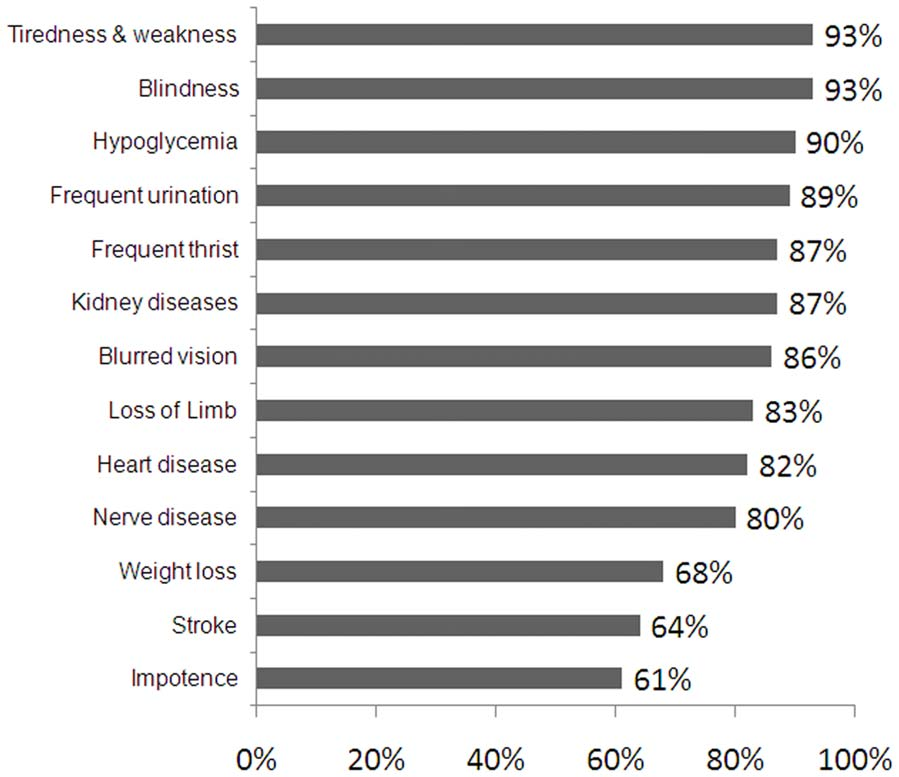
Diabetes General Knowledge (n = 575).

**Figure 2:**
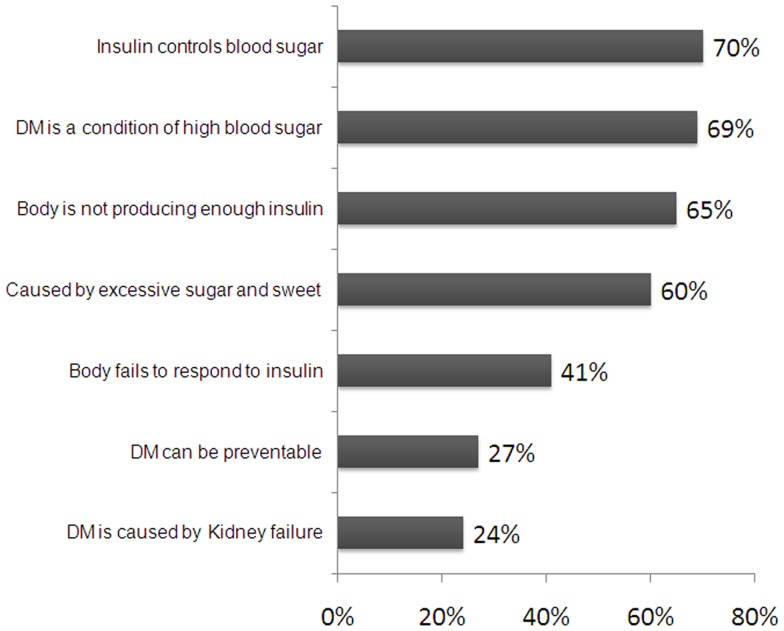
Knowledge of Diabetes Symptoms and Complications(n = 575).

The mean knowledge score was 15.7 (4.4), which fall within our definition of ‘Poor Knowledge’. Cronbach's alpha for the knowledge score was 0.674 and all items, except knowledge about impotence, were positively correlated with total score. In fact, 33% had ‘good knowledge’, 36% had ‘fair knowledge’, and 31% had ‘poor knowledge’. Percentages of correct answers to questions on general DM knowledge and on DM symptoms and complications are shown in Figures 1 and 2 respectively.

Most (89%) of the surveyed patients had seen a diabetic educator since their diagnosis, but many only a few times. Most patients (87%) cited doctors as the primary source of DM knowledge, but other sources were also frequently mentioned (Table 3).

**Table 3:** Sources of DM Knowledge among the Study Participants (n = 575).

Source of DM Health Information (can choose more than one)	N	% (95% C.I.)
Doctors	494	87.4 (84.7–90.1)
Nurses	167	31.0 (27.1–34.9)
Pharmacists	11	2.1(0.9–3.3)
Electronic media	218	41.0 (36.8–45.2)
Health educator	161	30.5 (26.6–34.4)
Dieticians	106	20.1 (16.7–23.5)
Friends and family	179	33.3 (29.3–37.3)
Published media	142	26.7 (22.9–30.5)
**Frequency of seeing diabetes educator**		
None	30	11.5 (7.6–15.4)
Once	68	26 (20.7–31.3)
Twice	50	19 (14.2–23.8)
More	114	44 (38.0–50.0)

Knowledge of diabetes varied significantly among nationalities, with Asians (mostly Indians and Pakistanis) having a higher mean knowledge score than UAE citizens, other Arab nationalities, and patients from the Gulf Council Countries. Other factors affecting diabetes knowledge were sex, age, level of education, marital status, profession, income, insulin treatment, mode of diagnosis and duration of diabetes (Table 4). Interestingly, analysis showed a positive correlation between patients' knowledge and the number of contacts with a diabetic education in the last two years.

**Table 4:** Mean Diabetes Knowledge, Attitudes and Practice Scores for Different Characteristics of the Participants (n = 575).

Variable		Total know Score	Total Practice Score	Total Attitude score	HbA1c
		Mean	Mean	Mean	Mean
**Sex**	Male	17.08	24.16	27.32	7.62
	Female	15.26	23.55	27.81	7.68
	*p. value*	0.000	0.273	0.441	0.891
**Age group**	< = 39 years	16.79	23.97	27.30	8.04
	40–49 years	16.52	23.85	28.32	7.70
	50–59 years	16.37	24.46	27.75	7.36
	60> =	14.98	23.20	27.04	7.84
	*p. value*	0.001	0.386	0.501	0.769
**Marital status**	Single	16.81	25.17	28.85	8.00
	Married	16.43	24.08	27.66	7.71
	Divorced	14.80	21.15	27.80	7.72
	Widowed	14.13	22.19	26.39	7.24
	*p. value*	0.000	0.011	0.322	0.883
**Nationality**	UAE	15.70	23.87	27.81	7.84
	GCC	15.48	22.95	26.75	7.45
	Other Arabs	17.73	24.23	27.62	7.54
	Asians	19.50	29.00	21.00	.
	*p. value*	0.000	0.342	0.515	0.759
**Occupation**	Gov. employed	18.17	24.83	27.87	7.03
	Private employee	18.42	24.29	30.08	7.79
	Retired	16.46	23.94	26.74	7.12
	Housewife	14.97	23.34	27.88	7.98
	Private business	19.25	23.75	32.25	11.00
	Others	16.11	25.81	27.42	8.54
**Level of education**	*p. value*	0.000	0.181	0.293	0.448
	Illiterate	14.74	23.31	27.65	7.92
	Primary school	16.59	24.34	27.44	7.93
	Secondary school	17.27	23.78	28.40	7.38
	University	19.48	26.05	27.16	6.31
	Post graduate	19.67	25.00	30.67	5.30
	*p. value*	0.000	0.053	0.762	0.285
**Monthly family income**	Less than 5000	15.84	23.36	26.75	7.44
	5000–9999	16.27	24.24	28.38	7.77
	10000–15000	15.77	23.73	27.75	8.26
	More than 15000	18.26	25.94	28.66	6.62
	*p. value*	0.008	0.133	0.127	0.570
**Mode of DM diagnosis**	Incidental	17.08	24.91	28.44	7.14
	Symptomatic	15.76	23.43	27.38	7.97
	*p. value*	0.000	0.009	0.111	0.124
**Insulin treatment**	Yes	16.20	24.27	27.32	7.47
	No	15.00	21.64	30.18	7.65
	*p. value*	0.000	0.189	0.003	0.752
**Frequency of seeing diabetes educator in the past 2 years**	*None*	15.74	23.29	28.31	7.98
	*Once*	14.65	24.47	28.41	7.45
	*Twice*	18.00	24.92	27.62	7.38
	*More than twice*	17.50	25.79	26.70	5.71
	*p. value*	0.000	0.001	0.187	0.020
**Duration of diabetes**	*One year or less*	14.70	21.40	27.32	8.71
	*1.1–5 years*	15.93	24.00	28.22	7.82
	*5.1–10 years*	16.10	23.83	27.62	7.17
	*10.1–20 years*	16.46	24.60	27.57	7.93
	*p. value (trend)*	0.003	0.007	0.399	0.740

### Assessment of Attitudes

Cronbach's alpha of the attitude score was 0.845 and all items were positively correlated with the overall score. Analysis showed that the majority of patients (72%) had a negative attitude towards having diabetes. However, only 6% expressed a ‘negative attitude’ towards the importance of DM care (Table 5), notably of controlling blood sugar levels and body weight, as well as compliance with medications. Bivariate analysis showed that the only factor that is associated with attitude is the type of DM (Table 4).

**Table 5:** Attitudes towards DM and DM Care among the Study Participants (n = 575).

Attitudes towards having DM	N (%)
Positive attitude	157 (28)
Negative attitude	410 (72)
**Attitudes towards the importance of DM care**	
Positive attitude	559 (94)
Negative attitude	36 (6)

### Assessment of Patients Practice towards DM Control

Analysis showed that most patients had satisfactory practice, and that the majority had reported regular routine follow up (Table 6). A large minority however, did not follow a diet, or control their weight. Also a substantial proportion was not exercising and admitted lack of compliance to medications Reported blood sugar control and monitoring were generally poor (Table 6). Only 27% of patients had good glycemic control.

**Table 6:** Diabetes Practices of the Study Participants (n = 575).

Variable	N	% (95% C.I.)
**DM practice scores levels**		
Good practice	217	37.7 (33.7–41.7)
Satisfactory practice	270	47.0 (42.9–51.1)
Poor practice	88	15.3 (12.1–17.9)
**Patients' control of DM**		
Always attending DM clinic for follow-up	452	80.4 (77.2–83.6)
Never controlling weight	93	17 (13.9–20.1)
Not undertaking any physical exercise	95	16.6 (13.6–19.6)
Not following any special DM diet	158	27.7 (24.0–31.6)
Not complying with medication	55	9.8 (7.4–12.2)
Never checked or cared for toes and feet	103	18.1 (15.0–21.2)
Never taken care when cutting toe nails	65	11.5 (8.9–14.1)
**Patients' self control of blood sugar**		
Always in good control	97	17.1 (14.0–20.2)
Often in good control	223	39.3 (35.3–43.3)
Sometimes in good control	195	34.4 (30.5–38.3)
Never in good control	52	9.2 (6.8–11.6)
**Patients' self test of blood sugar**		
Always test for blood sugar	235	41.7 (37.7–45.7)
Often check for blood sugar	126	22.4 (19.0–25.8)
Sometimes take blood sugar test	67	11.9 (9.3–14.5)
Never took blood sugar test	135	24 (20.5–27.5)
**Barriers of self testing among DM patients**		
Too expensive	52	10.2 (7.7–12.7)
Too painful	7	1.4 (0.4–2.9)
Not really needed	43	8.4 (6.1–10.7)
Don't know how to read results	24	4.7 (3.0–6.4)

Bivariate analysis showed (marginally) significant associations between the practice score and level of education, marital status, mode of diagnosis, duration of disease, insulin use and frequency of seeing diabetes educator (Table 4). There were no statistically significant association between patients' practice score and family history of DM, sex, age, nationality, monthly income or occupation (Table 4).

There was a weak, but statistically significant, correlation between the level of knowledge and practice and also between attitudes and practice (r = 0.320, p<0.001 and r = 0.270, p<0.001, respectively). Similarly there was a weak, but statistically significant association between knowledge and attitude scores (r = 0.115, p = 0.006). HbA**_1c_** was not statistically significantly correlated with any of the three scores.

### Multivariate Analysis

Stepwise linear regression for the total knowledge scores, total practice scores, and total attitudes scores on covariates identified in bivariate analysis showed several significant (adjusted) associations. Table 7 shows the results for the knowledge score and Table 8 for the practice score. No variables were identified as significantly predictive of the attitude score in this regression analysis. Regression analysis, using HbA**_1c_** as a dependent variable and the covariates of age, sex, level of education, nationality (UAE or not) , type of DM, and marital status (married or not) as independent variables showed that only the level of education (as continuous variable) and type of DM were (negatively with level of education) independently associated with HbA**_1c_** levels (Table 9).

**Table 7:** Patients Characteristics associated with Diabetes Knowledge Score in stepwise linear regression (n = 575).

Model	Un -standardized Coefficients		Sig.
	B	Std. Error	
(Constant)	14.996	.905	.000
Level of education	1.210	.164	.000
Gender (Male)	1.026	.325	.002
Type of DM	−1.014	.340	.003
Married	1.260	.354	.000
Family history of DM	−.828	.331	.011
UAE nationality	.716	.328	.029
Duration of DM	.061	.022	.007
Freq. of seeing diabetes educator	.256	.098	.009

*Dependent variable: Total Knowledge Score. Co variables entered were: level of education, gender, age, type of DM (insulin treated/not on insulin), married, frequency of seeing a DM educator in the past 2 years, duration of DM, UAE nationality, family income, mode of diagnosis, family history of DM, being employed.*

**Table 8:** Patients characteristics associated with Practice Score in stepwise linear regression (n = 575).

Model	Un -standardized Coefficients		Sig.
	B	Std. Error	
(Constant)	20.081	.865	.000
Married	1.250	.617	.043
DM duration	0.096	.039	.014
Level of education	0.732	.280	.009
Freq. of seeing diabetes educator	0.541	.173	.002

*Dependent Variable: Total Practice Scores. Co variables entered were: level of education, sex, age, type of DM (insulin treated/not on insulin), married, frequency of seeing a DM educator in the past 2 years, duration of DM, UAE nationality, family income, mode of diagnosis, family history of DM, being employed.*

**Table 9:** Patients Characteristics associated with Glycemic Control in stepwise linear regression (n = 575).

Model	Un -standardized Coefficients		Sig.
	B	Std. Error	
(Constant)	10.484	.966	.000
Type of DM	*−1.169*	**.** *494*	.019
Level of education	−.448	.219	.042

*Dependent Variable: HbA* ***_1C._*** *.Co variables entered: age, sex, level of education, UAE nationality, type of DM (insulin treated/not on insulin), married (0/1).*

## Discussion

Studies from both developed and developing countries have reported that diabetes knowledge is generally poor among diabetic patients [4]–[7], [14]–[17]. However, it is difficult to compare our results with others, as most of the studies used different instruments and/or are carried out among different ethnic or age groups. This study shows that the levels of knowledge seemed particularly low in the UAE. For example, two thirds of our patients cited excessive sugar consumption as the primary cause of the disease, while less than one third was aware that type 2 diabetes can be prevented or delayed. However, patients' general awareness of diabetes symptoms and complications was relatively high, perhaps because they had experienced these symptoms themselves or observed them in fellow-patients. We observed several correlates of knowledge, attitudes and practice. Some of our findings, e.g. that men had higher mean knowledge score than women appear to conflict with other studies [17], [18], [19]. Other correlates, such as the effects of education, are predictable. Of all significant correlates of knowledge and practice, education is the only modifiable risk factor. Fortunately, education is now practically universal in the UAE, and illiteracy is expected to disappear gradually.

Our study also shows that a history of diabetes in first degree relatives has a positive impact on diabetes knowledge. Having a close relative with chronic disease may be a good source of health information [20], [21], but such informal sources cannot be relied upon.

A major point to address therefore is regular access to/contact with diabetic educators which currently is severely substandard. However, while improved knowledge would definitely facilitate patient management, it would not necessarily guarantee improvement in the overall outcomes. This study showed no correlation between the level of knowledge and glycemic control, while other studies reported conflicting findings [22], [23], [24]. It is therefore essential to direct more resources to improving both the knowledge of diabetic patients, and the development of innovative tools and educational models that improve patient's compliance and practices. Such efforts would require further in-depth research on diabetic patients' knowledge, attitudes and practices and how they interrelate.

As our study was outpatient hospital based, the results may not be truly representative of all DM patients in the UAE. In particular, the fact that the study was conducted in university teaching hospitals, where diabetes education may be more readily accessible to patients, raises concerns that diabetic patients attending primary health care centers in the region with less access to diabetes education may have even poorer diabetes awareness and practices. The results suggest that special attention and increased care are required for the elderly diabetic patients in the UAE who are mostly illiterate. Also, patients on insulin should receive special attention as knowledge of DM management for them is a key.
